# The Stroma—A Key Regulator in Prostate Function and Malignancy

**DOI:** 10.3390/cancers4020531

**Published:** 2012-05-29

**Authors:** Christina Hägglöf, Anders Bergh

**Affiliations:** Department of Medical Biosciences, Pathology, Umeå University, Umeå 90185, Sweden

**Keywords:** prostate, cancer, metastasis, microenvironment, stroma

## Abstract

Prostate cancer is a very common and highly unpredictable form of cancer. Whereas many prostate cancers are slow growing and could be left without treatment, others are very aggressive. Additionally, today there is no curative treatment for prostate cancer patients with local or distant metastasis. Identification of new, improved prognostic and diagnostic biomarkers for prostate cancer and the finding of better treatment strategies for metastatic prostate cancer is therefore highly warranted. Interactions between epithelium and stroma are known to be important already during prostate development and this interplay is critical also in development, progression of primary tumors and growth of metastases. It is therefore reasonable to expect that future biomarkers and therapeutic targets can be identified in the prostate tumor and metastasis stroma and this possibility should be further explored.

## 1. Introduction

Prostate cancer is the most common form of malignancy among men and the risk of dying in this disease is particularly high in Sweden. Approximately half of the patients are over 70 years of age at diagnosis. Prostate cancer is commonly diagnosed after finding elevated levels of prostate specific antigen (PSA) in blood. PSA screening and early treatment reduce mortality in prostate cancer [1]. Many prostate cancers are however indolent and do not need active treatment. Unfortunately, current diagnostic procedures do not safely discriminate indolent tumors from the life threatening form of the disease. Screening and early treatment are therefore associated with a substantial risk with overtreatment. We therefore need to improve: (1) our methods to diagnose and prognosticate prostate cancer, (2) our capability to treat aggressive prostate cancer. Aggressive cancer generally metastasizes to the bone, is difficult to treat, and is the ultimate cause of death for prostate cancer patients. In this review we argue that increased knowledge on the prostate tumor and metastasis stoma may help us solve both these problems.

For many years cancer research was essentially focused on characterization of the “tumor cells”, but with time, also the other components of a tumor than the “tumor cells” have been established as important factors for tumor development, growth, metastasis and metastasis growth [2,3,4]. In addition to cancer epithelial cells, a primary tumor or metastasis consist of e.g., blood vessels, lymph vessels, cancer associated fibroblasts (CAFs), immune cells, nerves and extracellular matrix (ECM). These non-epithelial components of a cancer are often collectively referred to as the tumor stroma. The emerging concept that the stroma is of major importance in tumor biology is not a surprise to researchers working with prostate cancer, because in this organ it is already firmly established that the development and overall function of the normal prostate is dependent on a hormonally regulated crosstalk between epithelium and stroma [5]. In addition, pioneering studies performed years ago showed the importance of stromal cells in facilitating growth of primary tumors and metastases. More recent studies also suggest that the tumor stroma is a valid target for therapy and that stroma factors could serve as prognostic and treatment predictive markers.

## 2. The Stroma Regulates Normal Prostate Development and Function

The prostate is derived from the embryonic urogenital sinus under the influence of androgens. This process requires interactions between stromal and epithelial cells, more specifically the endodermal epithelium of the urogenital sinus (UGE) and the mesodermal urogenital sinus mesenchyme (UGM). Prostate development is initiated by androgen stimulation of the UGM, which in turn leads to differentiation of the prostatic epithelium. If the UGE and UGM are separated, prostate development will not take place. Tissue recombination experiments have also shown that the UGM has the potential to differentiate epithelium from other organs into prostate epithelium. The differentiation of UGM is equally dependent on interactions with prostate epithelium, and the epithelium is necessary for smooth muscle differentiation of the UGM [6]. Hence, prostatic development, resulting in ductal trees and lobes with secretory epithelium and a stroma consisting mainly of smooth muscle cells, can only take place when UGM and UGE can interact in the presence of androgens.

In the adult, prostate function is regulated by androgens and estrogens and both these steroid hormones act through primary effects in the stroma and epithelium. In the adult prostate, androgen receptor (AR) positive cells in the stroma regulate epithelial cell growth, death and differentiation via stroma-produced “andromedins”. For example members of the fibroblast growth factor (FGF), insulin-like growth factor (IGF), epidermal growth factor (EGF), Wnt and hepatocyte growth factor (HGF) families apparently function as andromedins [7,8]. ARs in luminal epithelial cells maintain cell survival whereas AR in basal/intermediate epithelial cells suppress proliferation [9,10,11,12,13]. Estrogen receptor (ER) α is expressed in the prostate stoma and mediates secretion of stroma factors stimulating epithelial cells. ERβ is expressed in epithelial cells and mediates inhibitory and differentiating functions [14].

Castration-induced normal prostate shrinkage (the standard treatment for prostate cancer) is in part dependent on actions in AR expressing cells in the prostate stroma [15]. In prostates lacking ARs in the stroma only a blunted castration response is seen, but castration-induced prostate involution is more unaffected if epithelial AR are depleted [7].

Castration-induced prostate glandular shrinkage is preceded by a vascular involution and reduced blood flow [16,17,18] suggesting that the subsequent epithelial involution is caused by hypoxia [19,20,21]. Testosterone stimulated prostate growth is in turn dependent on vascular endothelial growth factor (VEGF) and angiopoietin-driven angiogenesis [22,23] and accumulation of inflammatory cells secreting factors potentiating epithelial growth and differentiation [24,25].

Castration-induced prostate shrinkage is also dependent on transforming growth factor receptor beta II (TGFβRII) in the prostate stroma. Local differences in stroma composition and function along individual prostate ducts determine epithelial androgen dependency. The luminal epithelial cells in ducts adjacent to the urethra are normally castration resistant as they are protected from apoptosis by high constitutive secretion of Wnt ligands from the adjacent stroma [26,27]. In contrast, luminal cells in the more distal parts of the ducts undergo apoptosis as a result of TGFβ signaling in the adjacent stroma.

## 3. The Prostate Stroma Is Heterogeneous and Affected by Age and Non-Malignant Diseases

In addition to local differences in stroma morphology and function along individual prostate ducts (see above), there are also differences in morphology, gene expression pattern and androgen dependency in the different prostate lobes in rodents [7]. Similarly, the stroma in the different zones of the human prostate demonstrates differences in gene expression [28]. Such differences may explain why cancer originates more commonly in the peripheral than in the transitional zone of the prostate [29].

Benign prostatic hyperplasia (BPH), a very common disease, is largely caused by altered stroma cell function resulting in stroma and epithelial cell growth [30]. Stromal cells from the normal peripheral zone, benign prostatic hyperplasia (BPH), and cancer have different effects on prostate epithelial cells. Stromal cells from the normal peripheral zone lack the capacity to induce growth, whereas BPH stroma give rise to grafts with a benign appearance. However, prostate epithelial cells combined with cancer associated stroma forms grafts that are fast growing and have a more aggressive appearance [31].

The prostate stroma is also affected by ageing. Inflammatory cells become more abundant and stromal fibroblasts become senescent. These senescent fibroblasts are less dependent on androgens [32] and particularly effective in stimulating prostate cancer cells *in vitro* [33]. The proportion of myofibroblasts is also increased with age, and stroma isolated from older individuals display a different gene expression profile as compared to stroma from younger individuals [34]. Further studies are needed to explore if the clear age dependency of prostate cancer is related to stroma ageing.

## 4. Prostate Cancer Development Is Characterized by Co-Development of Epithelial and Stromal Changes

As outlined above alterations in one prostate compartment, due to the close functional coupling between the epithelium and the stroma, are expected to also result in alterations in the other. During prostate cancer development, changes in the epithelium therefore causes changes among the different cell types present in the stroma. These stroma alterations, in turn, affect the epithelium. For example, in a mouse model of prostate cancer, pRb inhibition in tumor cells was shown to cause a stromal upregulation of p53. This triggered the selection and expansion of a population of p53-null stromal fibroblasts, which in turn supported epithelial loss of p53 and tumor progression [35]. In addition, tissue recombinations where UGM or/and UGE have been transformed with Myc and Ras, showed that oncogenic introduction into both tissue compartments is required for carcinoma development [36]. Moreover, a number of studies where prostate cancer or epithelial cells have been combined with fibroblasts and injected in immunocompromised mice show how stromal cells affect the tumorigenicity of epithelial cells. For example, combining prostate cancer cells with fibroblasts before injection into mice enhanced tumor growth and incidence [37].

Given the functional coupling between the stroma and epithelium it is not surprising that the magnitude of stroma changes are related to tumor aggressiveness and patient outcome (Table 1), and that prostate cancer development is associated with profound changes in stroma gene and protein expression.

**Table 1:** Factors associated with prognosis or tumor grade in prostate cancer stroma.

	Factor	Alteration associated with poor prognosis/tumor grade	Ref.
**General morphology**	Reactive stroma grade	Increase	[38]
	Gleason score *	Increase	[39]
**Cellular composition**	Macrophages	Increase	[40]
	Mast cells	Decrease	[41]
	T-cells	Increase	[42]
	CAFs	Increase	[43]
	Vascular density	Increase	[44]
	Smooth muscle cells	Decrease	[45]
**ECM proteins**	HA	Increase	[46]
	COL1A1	Increase	[38]
	VCAN	Increase	[47]
	POSTN	Increase	[48]
	MMP9	Increase	[49]
**Growth factor receptors**	AR	Decrease	[50]
	PDGFRβ	Increase	[51]
	TGFβRII	Decrease	[26]
**Others**	TRAIL	Increase	[52]
	PAR-1	Increase	[53]
	CAV-1	Decrease	[54]
	EpCAM	Increased	[55]
	CDH11	Increased	[56]

* Gleason score is generally defined as the grading of glandular pattern but could equally well be defined as grading of stroma pattern.

### 4.1. Alterations in Stroma Gene and Protein Expression Pattern

The stromal gene expression pattern is altered during prostate cancer progression and the magnitude of these changes can be used to predict tumor aggressiveness [57,58,59]. As the cellular composition of the stroma is markedly changed in tumor *vs*. non-malignant stroma (see below) the contribution of the individual stoma cell types for the overall changes in gene and protein expression in prostate cancer stroma is largely unknown. Stromal gene expression is prognostic and treatment predictive in breast cancer, but importantly the stroma gene expression pattern characterizing breast and prostate cancer are largely different, suggesting that the stroma response is tumor-type specific [57].

### 4.2. Smooth Muscle Cells and CAFs

Adult prostate stroma normally consists mainly of smooth muscle cells. During carcinogenesis, the smooth muscle cells are gradually replaced by fibroblasts, often termed cancer-associated fibroblasts (CAFs). Consequently the cancer stoma is characterized by decreased expression of smooth muscle cell markers such as desmin and smooth muscle actin, and increased fibroblast markers such as vimentin.

CAFs are phenotypically and functionally different from normal fibroblasts and are in many ways similar to the fibroblasts in wound healing and fibrosis. The gene and protein expression pattern in prostate CAFs is also similar to that in the stroma during normal prostate development and the biological processes regulating glandular morphogenesis [60]. Whether CAFs are generated by activation of already present fibroblasts and pericytes, or recruited from the bone marrow, or as a result of transdifferentiation of epithelial or endothelial cells is not yet clear [61,62,63,64,65], and different CAF subpopulations could have different origins. CAFs consist of a heterogeneous collection of cells expressing different markers e.g., fibroblast activating protein (FAP), fibroblast specific protein (FSP), alpha smooth muscle actin (αSMA), and platelet derived growth factor (PDGF) receptors [61]. CAF subpopulations isolated from prostate cancer patients have specific biochemical characteristics, possibly as a result of localized influences from adjacent heterogeneous populations of tumor epithelial cells, and some CAF subtypes are more tumorigenic than others [66,67]. CAF heterogeneity, where different CAF subtypes contribute with different stimulatory factors, is actually a key element in their overall tumor promoting activity [68,69].

CAFs are known to be able to affect cancer cells and other the tumor compartments e.g., by secreting growth factors, ECM components and proteases. Prostate CAFs are e.g., characterized by high secretion of TGFβ, stromal cell-derived factor 1 (SDF-1), CXCL14, hypoxia inducible factor 1 alpha (HIF1α), GFRα, ERα, Hes1, VEGF-D, asporin (ASPN), IGF-1, tenascin-C (TNC), collagen 1 (COL1A1), EGF, FGF2, FGF7, HGF and αSMA. In contrast, prostate CAFs displays a reduced production of caveolin-1 (CAV-1), S100A6, natural killer tumor recognition sequence (NKTR) and stanniocalcin-1 (STC1) [60,67].

Notably, a normal stroma can inhibit carcinogenesis. When rat prostate cancer tumors were grown in its corresponding tumor stroma it resulted in undifferentiated tumors. However, if the original tumor stroma was replaced with UGM or seminal vesicle mesenchyme, this gave rise to highly differentiated, well-organized structures with decreased growth rate [70].

### 4.3. Blood and Lymph Vessels

Prostate cancer development is associated with increased secretion of factors stimulating formation of new blood and lymph vessels and decreased secretion of inhibitors [17]. Some of the regulators of vascular growth are produced in the tumor epithelium and others in the tumor stroma. The density and morphology of blood and lymph vessels are both prognostic marker, *i.e*., the more vessels the poorer the outcome (Table 1) [44]. Inhibition of angiogenesis also retards prostate cancer growth, at least in experimental models.

### 4.4. Inflammatory Cells

Prostate cancer development is associated with an influx of macrophages, lymphocytes and mast cells to the normal tissue adjacent to the tumor [71] and into the tumor stroma. These inflammatory cells may by secreting various cytokines have stimulatory or inhibitory effects on adjacent CAFs, blood vessels and tumor epithelial cells [72]. The magnitude of these inflammatory processes is related to tumor aggressiveness (Table 1) and in experimental models inhibition of mast cells and macrophages reduce tumor growth [41,73].

Notably, castration treatment results in an additional influx of inflammatory cells, like macrophages, mast cells and lymphocytes, into prostate tumors or to the invasive front [41,74], where they may stimulate tumor growth. For example, accumulating B-lymphocytes secrete factors like lymphotoxin B that stimulate tumor epithelial cells and thus promote castration resistant tumor growth [74].

### 4.5. Extracellular Matrix

Prostate cancer development is associated with an altered composition of the extracellular matrix. COL1A1, hyaluronan (HA), versican (VCAN), periostin (POSTN) and matrix metalloproteinase (MMP) 9 are all increased and the magnitude of these responses are related to tumor aggressiveness (Table 1). Some of these EMC components are clearly produced by CAFs whereas others could also be produced by tumor infiltrating macrophages. Manipulation of the matrix, for example increasing or decreasing HA, influences tumor growth [46].

### 4.6. How Do the Different Components of a Prostate Cancer Stoma Interact with Each Other?

Most studies on the tumor stroma examine how individual components of the stroma interact with cancer epithelial cells, or how cancer epithelial cells interact with individual stroma components. Studies examining how the different stroma cell types and matrix interacts with each other are largely lacking but it would be highly surprising if CAFs, ECM, inflammatory cells and blood vessels did not influence each other in ways of importance for overall tumor growth and spread.

## 5. Prostate Cancer Could Be the Result of Primary Changes in the Stroma

Prostate cancer is generally assumed to originate in premalignant changes in the epithelium gradually progressing to carcinoma *in-situ* and cancer. Bhowmick *et al*. however observed that knocking out the TGFβRII in a subset of prostate fibroblasts resulted in prostate neoplasia, demonstrating that a primary effect in stroma TGFβ signaling can cause cancer in this organ [75]. Loss of TGFβRII is seen in a subset of tumor stroma cells in patients [26], and both in experimental models [68,69] and in patients this seems to promote the appearance of aggressive prostate cancer. Interestingly, the most aggressive tumors are formed when the stroma is composed of a mixture of CAFs lacking TGFβ signaling and others where it is unaffected. Subsets of CAFs apparently cooperate and produce different factors of importance for tumor progression and growth [68,69]. Stroma TGFβRII is also required for castration induced prostate regression indicating that castration resistance can be caused by stroma changes.

## 6. What Are the Signals Causing the Formation of a Prostate Cancer Stroma?

Several lines of evidence suggest that the formation of a tumor stroma, like a similar biological response *i.e*., the formation of a wound healing stroma is induced, at least in part, by the secretion of TGFβ. *In vitro,* TGFβ transform normal fibroblasts into CAF-like cells and such cells in turn stimulate tumor growth [67]. TGFβ is increased in prostate cancer epithelium and the magnitude of this is related to stroma angiogenesis and outcome in prostate cancer patients [76]. Stroma morphology is different in fusion-gene positive and negative tumors [77] suggesting that epithelial tumor cell phenotype affects adjacent stroma. TGFβ is however not the only factor causing development of a tumor stroma. Different growth factors and inflammation mediators produced by cancer cells and other cells in the tumor, such as, PDGFs, FGF-2 and hedgehog are apparently also involved in the activation of CAFs and other stroma components [7,61,78].

By implanting cancer cells into the normal rat prostate we recently showed that the presence of cancer induced adaptive changes not only in the tumor stroma but also in the surrounding normal prostate. We have named these phenomenon tumor instructed normal tissue (TINT), and in patient samples the magnitude of such TINT changes are related to tumor aggressiveness and patient outcome [46,51,71,79]. TINT changes can therefore be used for diagnostic and prognostic purposes and TINT may therefore also stand for tumor indicating normal tissue. Interestingly many of the changes in TINT are similar to those seen in the tumor stroma suggesting that the signals causing the formation of a tumor stroma extends far into the surrounding normal prostate [71].

## 7. The Stroma May Determine the Response to Treatment

Recent studies in other tumor types have suggested that stroma targeted therapies can be used to enhance the effect of standard therapies (which primarily targets tumor epithelial cells). As mentioned above it is possible that the standard therapy for prostate cancer, that is castration, is actually, in addition to the direct inhibitory effects of androgen shortage in tumor epithelial cells [80], also a stroma targeted therapy acting indirectly on the epithelium in two different ways: (1) castration inhibits the secretion of growth promoting factors from AR positive tumor stroma cells; (2) castration reduces blood flow (in the tumor and in the surrounding non-malignant prostate tissue) causing ischemic cell death among epithelial cells.

In patients the response to castration is most pronounced in non-malignant prostate tissue, moderate in primary prostate tumors and apparently more limited in hormone-naïve bone metastases [81]. Similar site-dependent (prostate *vs*. bone) effects of castration are seen also in experimental models where identical tumor cells are injected at different sites and treated [82]. The mechanisms explaining difference between tumor and normal prostate epithelial cells and site-specific effects of treatment in tumors are unknown but several mechanisms are possible. Changes within tumor epithelial cells could make them less dependent on circulating androgens, stroma-derived factors promoting cell survival and proliferation (that is a shift from endocrine-paracrine to autocrine regulation [80]) and more tolerant to hypoxia [20] than normal prostate epithelial cells. Prostate tumor development is however also associated with changes in stroma androgen-dependence [22,50]. Prostate cancer epithelial cells secrete factors inhibiting androgen action in stroma cells [83]. In line with this, stroma androgen receptors are reduced in primary prostate tumors and they are particularly low in aggressive cancers and metastases (Table 1, [84]). Consequently, patients with low AR levels in the stroma have a limited response to castration therapy [50]. One reason for this could be that stroma produced andromedins like IGF-1 are not, in contrast to the situation in normal prostate tissue, down-regulated by castration in such cancers [85]. It is therefore likely that stroma targeted therapies could enhance the effect of castration in such patients.

One effect of castration in the normal prostate is down-regulation of factors in the stroma causing vascular regression [86], but in contrast to the situation in the normal prostate castration does not appear to reduce blood flow in prostate tumors [16]. In a rat prostate cancer model, where castration in contrast to the situation in the epithelium failed to down-regulate stroma AR levels and vascular regulators such as PDGF-R and Tie-2, inhibition of these factors by additional treatments increased the effect of castration and resulted in reduced tumor growth, decreased vascular density and increased tumor cell apoptosis as compared to castration treatment alone [22].

Stromal TGFβRs are also nessescary for castration-induced prostate shrinkage [27] and loss of stroma TGFβRs, commonly seen in patients is related to castration resistance. Again suggesting that castration resistance could be due to changes in stroma responsiveness to regulatory signals. Stroma ERs are, in contrast, increased rather than decreased in prostate tumor stroma [57] indicating that the tumor stroma could be estrogen hyper-responsive.

Although most primary tumors and bone metastases initially responds to castration-therapy the disease generally relapses to castration-resistant prostate cancer (CRPC) growth. This is associated with increased local synthesis of androgens, appearance of constitutive active AR and increased AR signaling in tumor epithelial cells [87,88]. The role of the different stroma cell types, their secretory products and stroma AR in the development of CRPC in primary tumors and metastases is however largely unknown.

If the standard treatment for prostate cancer acts in part by affecting the tumor stroma, and as castration treatment is mainly used for and eventually fails in bone metastatic disease, if becomes extremely important to known whether the stroma is really similar in primary tumors and metastases.

## 8. Is the Stroma Similar in Primary Prostate Cancer and in Metastases?

Prostate cancer may metastasize to a variety of tissues and organs but metastases to bone are the most important ones clinically. Bone metastases are generally very painful and may cause skeletal complications such as fractures and spinal cord compression. They are often treatment resistant and are the main cause of death for prostate cancer patients.

The reason why prostate cancers preferably spread to and grow in the bone is unknown, but the ability to interact with cells in the microenvironment is crucial. To understand this microenvironment most researchers have examined interactions between prostate cancer cells and bone cells like osteoblasts and osteoclast [89,90,91], but as prostate cancer metastases primarily to bones with red bone marrow important interactions with blood forming cells and their stroma are also likely.

When prostate cancer cells arrive to for example a lymph node or to the red bone marrow they encounter a microenvironment that is highly different from that in the primary tumor. Although it is possible that metastatic cells may “bring their own soil”, *i.e*., arrive together with a few stroma cells from the primary tumor [92] the cancer cells need to instruct the new environment to form the stroma necessary to support colonization and growth. It is however also likely the microenvironment selects the cancer cells that are allowed to grow. It is thus not unlikely that a metastasis stroma shares characteristics with that in the primary tumor, but also that it in some aspect can be fundamentally different. The cell types used to create a metastasis stroma are largely undefined, but bone marrow stroma cells and mesenchymal stem cells present in a blood-forming bone marrow are probable sources.

Very few studies appear to have compared the stroma in paired primary tumors and metastatic lesions in prostate cancer. TGFβRII expression in CAFs is absent in human cancer bone metastases [93] and prostate cancer cells *in vitro* are able to suppress TGFβRII, smooth muscle actin and ECM production, and increase vimentin, integrins, MMP9 and MMP14 in bone marrow stroma cells [94] resulting in the formation of a more fibroblast like than myofibroblast-like stroma. When we examined prostate cancer bone metastases in patients the cancer cells were growing in a fibroblast like stroma [84] with less smooth muscle actin and AR expression and more SDF-1 expression than in the corresponding primary tumor stroma [77]. In addition, the number of tumor infiltrating macrophages was considerably larger in the metastases than in primary tumors. As stroma targeted therapies could be a novel way to enhance the efficacy of treatment directed towards tumor epithelial cells, the exact nature of the metastasis stroma therefore needs to be explored in more detail. If for example effects in AR and TGFβR positive stroma cells are of importance for mediating a full response to castration it should be noted that they are lacking in the bone metastasis stroma [84], and studies in patients (see above) and animal models of prostate cancer suggest that the response to castration is considerably more prominent in primary tumors than in metastases and that some metastases may actually be stimulated rather than inhibited by this type of treatment [95].

Many studies show associations between factors in the primary tumors and lymph node metastasis, but the actual stroma of prostate cancer lymph node metastasis is to our knowledge rather unexplored. Some studies however describe micro-environmental changes in lymph node stroma of other cancers. For example, analysis of cancer cell positive or negative lymph nodes from breast cancer patients revealed functional differences, including an increase in MMP proteolytic activity in tumor bearing lymph nodes [96]. Another recent study shows that lymph node stromal cells increased colorectal tumor formation and angiogenesis partly via SDF-1. In addition, lymph node stromal cells enhanced colon cancer cell drug resistance and the selection of a more aggressive colon cancer cell phenotype [97].

## 9. What Are the Key Factors Regulating the Formation of a Metastasis Stroma?

Also at the metastatic site, the cancer cells are likely to be dependent on interactions and co-development with stromal cells. Prostate cancer cells are able to produce a number of different factors known to influence bone physiology such as endothelin, BMPs, proteases, RANKL and PTHrP. The bone in turn provides prostate cancer cells with e.g., growth factors and cytokines (see [98] for more detailed information).

Bone is very rich in TGFβ and it is involved in bone resorption. It is therefore possible that TGFβ may serve a similar role in regulating stroma in both primary tumors and metastases. The role of TGFβ and its receptors in bone metastases is however largely unknown. Injection of TGFβR negative CAFs may promote growth of prostate cancer cell in the bone in comparison to TGFβR positive CAFs [93], but it is also reported that inhibition of TGFβ signaling decrease the growth of prostate cancer cells in bone [99].

The SDF-1/CXCR4 signaling pathway is important for prostate cancer metastasis to bone. Many prostate cancer cells express CXCR4 and osteoblasts, fibroblasts and endothelial cells in the bone marrow in turn express the ligand, SDF-1 [100,101,102,103]. Blocking of CXCR4 with a neutralizing antibody decrease prostate cancer cell invasivity, inhibits homing of prostate cancer cells to the bone and also attenuate growth of prostate cancer cells in bone [104].

Specific lipids have been shown to be another important factor for prostate cancer cells in the bone marrow, working both as a chemotactic element and as an energy source [105]. Interactions with adipocytes alter growth, morphology and gene expression of prostate cancer cells and depletion of adipocytes in the bone marrow strongly decrease the homing of prostate cancer cells to bone marrow in an aracidonic acid-dependent manner [106,107].

## 10. Conclusions

Work during the last decades has now firmly established that the development and function of the normal prostate, as well as the formation, growth and spread of prostate cancers are largely dependent on multidirectional interactions between different subtypes of prostate epithelial cells and their local microenvironments. Most of this knowledge has, however, been acquired by examining primary tumors, and studies on stroma-epithelial interactions in metastases are unfortunately largely lacking.

For several reasons the metastasis stroma must come more into focus in the future. For example: (1) metastasis to the bone marrow occurs early and is found in most prostate cancer patients already at diagnosis [108,109]. Fortunately most of these micro-metastases will remain dormant as a result of unknown micro-environmental influences at the metastatic site; (2) for prostate cancer truly localized to the prostate we already have excellent treatments such as surgery. What we lack are effective treatments for metastatic disease. To improve therapy for these men we need develop novel ways to target both the metastatic cells and their micro-environment, and this should probably be done in ways somewhat different from those effective in primary tumors.
